# GLOBathy, the global lakes bathymetry dataset

**DOI:** 10.1038/s41597-022-01132-9

**Published:** 2022-02-03

**Authors:** Bahram Khazaei, Laura K. Read, Matthew Casali, Kevin M. Sampson, David N. Yates

**Affiliations:** Research Applications Laboratory, National Center for Atmospheric Research, Boulder, CO 80301 USA

**Keywords:** Limnology, Hydrology, Geophysics, Geomorphology, Civil engineering

## Abstract

Waterbodies (natural lakes and reservoirs) are a critical part of a watershed’s ecological and hydrological balance, and in many cases dictate the downstream river flows either through natural attenuation or through managed controls. Investigating waterbody dynamics relies primarily on understanding their morphology and geophysical characteristics that are primarily defined by bathymetry. Bathymetric conditions define stage-storage relationships and circulation/transport processes in waterbodies. Yet many studies oversimplify these mechanisms due to unavailability of the bathymetric data. We developed a novel GLObal Bathymetric (GLOBathy) dataset of 1.4+ million waterbodies to align with the well-established global dataset, HydroLAKES. GLOBathy uses a GIS-based framework to generate bathymetric maps based on the waterbody maximum depth estimates and HydroLAKES geometric/geophysical attributes of the waterbodies. The maximum depth estimates are validated at 1,503 waterbodies, making use of several observed data sources. We also provide estimations for head-Area-Volume (*h-A-V*) relationships of the HydroLAKES waterbodies, driven from the bathymetric maps of the GLOBathy dataset. The *h-A-V* relationships provide essential information for water balance and hydrological studies of global waterbody systems.

## Abstract (summary)

**Table:** 

Measurement(s)	lake depth • reservoir depth • bathymetry • Head-Area-Volume relationship
Technology Type(s)	machine learning • Geographic Information System • bathymetry data processing
Sample Characteristic - Environment	water body
Sample Characteristic - Location	global

Machine-accessible metadata file describing the reported data: 10.6084/m9.figshare.16695070

## Background & Summary

The majority of Earth’s accessible fresh surface water is stored in more than 100 million lakes and reservoirs (hereafter waterbodies), which serve as vital resources for an exhaustive list of critical ecosystem functions and human and animal habitats^1^. Changes in storage volume and/or the timing due to climate variability, human activity, etc., can lead to disruptions of natural physiologic processes and affect water quality and quantity. Such changes and their negative consequences have been observed in waterbodies around the world (e.g., in the Aral Sea and Lake Erie), for which a scientific consensus on the climatological and hydrological drivers behind these associated changes in water storage is still evolving^2,3^. Ultimately, in order to understand these drivers of change, we need models built with accurate and detailed representations of waterbody physical characteristics.

Due to a lack of bathymetric data, physical and hydrologic models that simulate historic and future waterbody dynamics are limited since they rely on data sources that are largely model-based^4,5^. Advancements in computing, Geographic Information Systems (GIS), remote sensing (RS), airborne LiDAR, and optical imaging have increased accessibility and fidelity of waterbody geometry parameters^6–8^, reducing the reliance on limited ground-based observations. For instance, these technologies have led to advancements in estimates of time-varying waterbody parameters such as surface area, volume, and discharges^5,9–11^ and RS-based data services provide daily estimates of changes in global waterbody surface levels such as Cooley *et al*.’s analysis of water levels in global waterbodies^12^, Global Reservoirs and Lakes Monitor (G-REALM; https://ipad.fas.usda.gov/cropexplorer/global_reservoir/), and Database for Hydrological Time Series of Inland Waters (DAHITI; https://dahiti.dgfi.tum.de/en/), inferred from relevant information offered by a suite of satellites, e.g., ICESat-2 (https://icesat-2.gsfc.nasa.gov/), Jason-2 (https://www.jpl.nasa.gov/missions/jason-2/), and TOPEX-POSEIDON (https://sealevel.jpl.nasa.gov/missions/topex-poseidon/summary/). Despite the progress made, such products have not yet been fully utilized in operational hydrologic models^13^ due to limited reliability of high-quality cloud-free imagery data and a number of other latency and resolution issues^11^. RS-based bathymetry development is also limited to penetration depth of satellite data in offshore and open-lake areas. In addition, further refinements might be required to address the potential errors in nearshore areas if satellite imagery data is obtained during high-turbidity and resuspension events where sediment concentrations are high or errors that mountain shadows could cause in mountainous regions^5,14^.

There are numerous global^15–17^ and local^18^ datasets that provide estimates of basic waterbody parameters such as average depth, shoreline length, surface area, volume, and other geophysical parameters. Table 1 provides a list and associated parameters for the main existing datasets worldwide. Although these datasets provide valuable information for basic hydrologic and limnological modeling applications, they lack the bathymetric information needed to accurately and/or realistically depict geophysical conditions in the global inland waterbody systems and support long-term modeling of physical and biogeochemical processes and water balance simulations at an adequate spatial resolution.

**Table 1:** Major global and local waterbodies datasets.

Dataset	Data Provider	Number of Waterbodies	Region	Main Products (not limited to)
G-REALM	USDA	340	Global	Name, Location, Dam and River Name, A, V, Vres, Davg, tr, Elev, WA, lat, lon
GLWD	Lehner and Doll (2004)	253,067	Global	Location, P, lat, lon, Wetland Information
HydroLAKES	Global HydroLAB	1,427,688	Global	Name, Location, P, A, V, Vres, Davg, Qavg, tr, Elev, S, WA, lat, lon
GRanD	GWSP	6,862	Global	Name, Location, Dam and River Name, DL, DH, A, Vres, Davg, Qavg, Elev, WA, lat, lon
GLCP	Meyer *et al*. (2020)	1,422,499	Global	Name, Location, Watershed information, PP, A, T, WP, lat, lon
ReGeom	Yigzaw *et al*. (2018)	6,824	Global	Name, Location, GS, A, V, Vres, Davg, DIMavg, DH, h-A-V, lat, lon
NHDPlus	USGS and USEPA	448,512	US	Name, Location, P, A, V, Davg, Dmax, Elev, lat, lon
RMD	Rodgers (2017)	3,828	US	Name, Location, Dam Name, P, A, Davg, Vres, H, Qmax, Qavg, Elev, WA
Texas Waterbodies	TWDB	121	TX, US	h-A-V relationships (observed, ground-based)

Waterbody parameters are abbreviated as: P = shoreline length, A = surface area, V = total volume, Vres = active waterbody volume, Davg = average depth, Dmax = maximum depth, Qavg = average discharge flowing through the waterbody, Qmax = maximum discharge flowing through the waterbody, tr = residence time, Elev = waterbody surface elevation, S = average slope around the waterbody, WA = waterbody watershed area, DL = dam length, DH = dam height, H = hydraulic height, WSE = water surface elevation, GS = approximated geometric shape, DIMavg = average waterbody dimensions, h-A-V = head-Area-Volume relationships, PP = total watershed precipitation, T = average watershed temperature, WP = watershed population, lat = latitude, lon = longitude.

Despite a few efforts to develop bathymetry datasets for inland waterbodies, work needs to be done to refine global underwater topography and address the deficiencies of existing datasets. ETOPO1 (https://www.ngdc.noaa.gov/mgg/global/), for instance, is a raster-based global bathymetry dataset suitable for global and large-scale studies, however, it does not resolve smaller waterbodies due to its coarse resolution (1 arc-minute). Digital Elevation Models (DEMs) such as MERIT DEM^19^, SRTM^20^, HydroSHEDS Hydrologically Conditioned DEM^21^, DEM-H^22^, and NASADEM^23^ have masked and flattened waterbodies due to difficulties of estimating bathymetry at these locations without considering the geophysical properties of these systems. Other regional bathymetry datasets such as the Bathybase (http://www.bathybase.org/) and those compiled by several states in the US (e.g., Texas and Minnesota waterbodies) are applicable in local studies, however, are not scalable for large-scale hydrological modeling. United States Geological Survey (USGS) also compiles the Reservoir Sedimentation Database (RESSED; https://water.usgs.gov/osw/ressed/) which is aimed to provide bathymetry surveys for waterbodies in the US, although the dataset includes outdated surveys and covers less than ~0.5% of the waterbodies in the US.

The main objective of this study is to present a new GLObal Bathymetric dataset, GLOBathy, which provides validated estimates of maximum depth (*Dmax*), bathymetric maps in resolution of 1 arc-second, and head-Area-Volume (*h-A-V*) relationships for 1.4+ million waterbodies originally obtained from the well-established HydroLAKES^4^ dataset (https://www.hydrosheds.org/pages/hydrolakes). GLOBathy is the first dataset to provide reliable estimates of maximum depth, bathymetry, and *h-A-V* relationships on such a scale and at high resolution, relevant to a wide range of hydrological, environmental, biological, limnological, and coastal applications.

## Methods

We utilize HydroLAKES as the dataset unit for GLOBathy because it provides the most comprehensive spatial coverage of waterbodies on Earth. HydroLAKES provides an exhaustive list of waterbody characteristics information including shoreline length, surface area, volume, average depth, average discharge, elevation, residence time, drainage area, and average slope around the waterbody for about 1.43 million waterbodies with global coverage, however, it does not supply critical geospatial bathymetric information. The workflow to create GLOBathy is summarized here and then described step-by-step. First, we tested a series of candidate functions to find the best form to estimate *Dmax* for the HydroLAKES dataset. The candidate functions were validated using a compiled set of *Dmax*, shoreline length (*P*), surface area (*A*), volume (*V*), waterbody surface elevation (*Elev*), and watershed area (*WA*) observations from 1,503 waterbodies around the world. After computing *Dmax* for all HydroLAKES, we calculated bathymetry by using the distance method developed (explained later in the article) by Hollister and Milstead^8^, borrowing attributes from HydroLAKES. As a final step, we developed *h-A-V* relationships for each waterbody based on the generated bathymetry and validated with available field observations.

### Estimation of maximum depth for HydroLAKES

Two different approaches were tested to compute *Dmax*. In the first method, we followed the assumption that waterbodies can be approximated by regular geometric shapes^17^ and calculated *Dmax* for four geometries: box (i.e., with vertical sidewalls), cone, triangular prism, and ellipsoid. *Dmax* was calculated for each shape given estimates of *A* and *V* and corresponding geometric functions. The second formulation assumed that *Dmax* is a function of the waterbody geometric and geophysical characteristics such as *P*, *A*, *V*, *Elev*, and *WA*. Many studies have validated this assumption^4,8,24–28^, and in particular, Heathcote *et al*. demonstrated the practicality of using geographic conditions to predict a waterbody’s *Dmax* and *V*^29^.

**Fig. 1:**
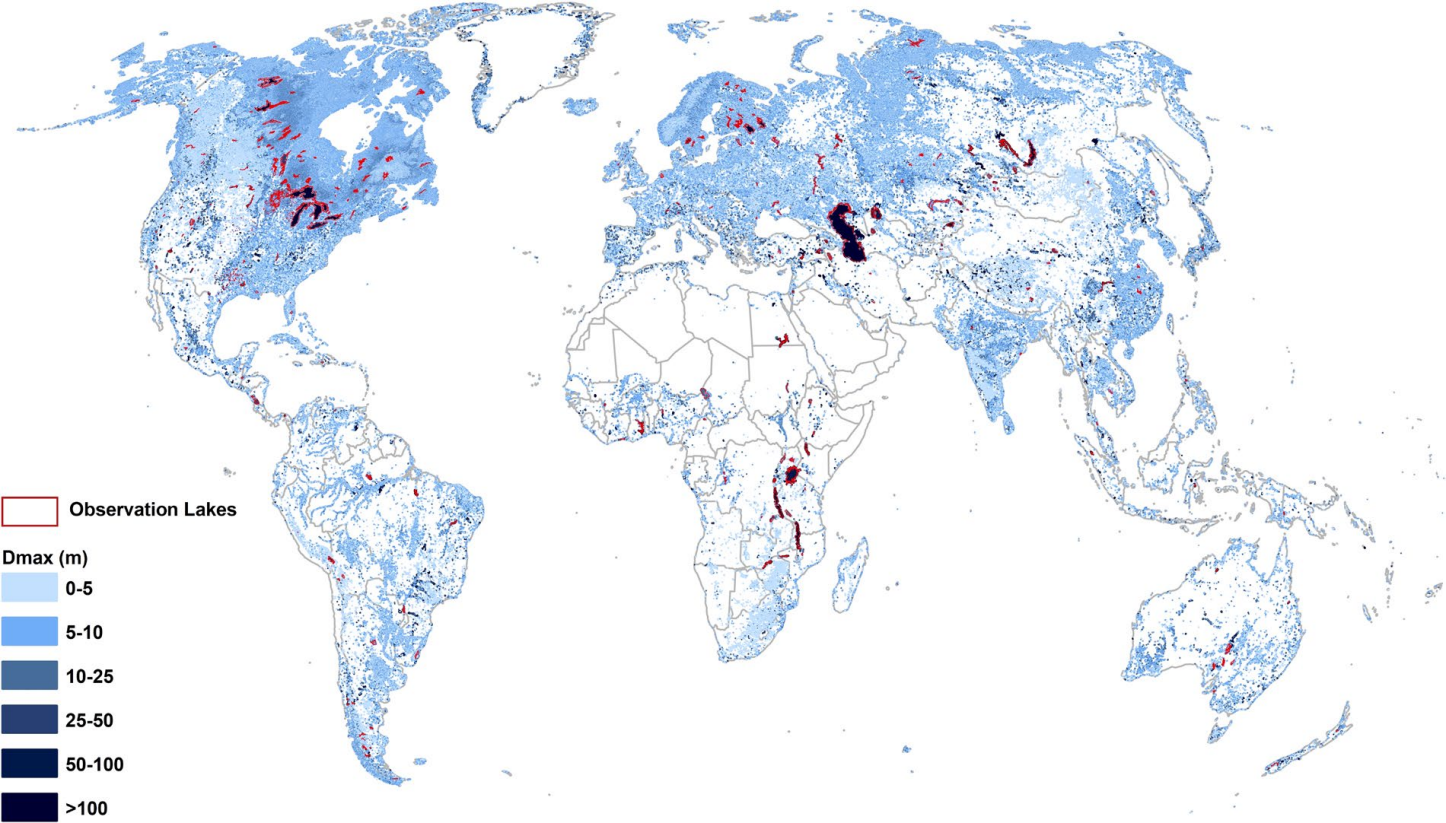
Global waterbodies maximum depth (Dmax) distribution. Observational waterbodies are shown with red polygons.

In the second approach to estimating *Dmax*, we tested several functional forms (i.e., exponential, multiple regression, etc.) and found after validating over 1,503 observations that the random forest regression is the best empirical model. Random forest is a learning method primarily for classification and regression by constructing an ensemble of decision trees, randomly and independently sampled from a feature space (i.e., a forest)^30,31^. To avoid overfitting of the regression model, we carried out a cross-validation. Best results were obtained when the number of trees were set to 30 in the random forest regression. Random forest regression also resulted in better estimates *of Dmax* than using the geometric shapes (as detailed in the Technical Validation section), therefore, we used this method to continue with generation of the bathymetry data. The validation dataset consists of several local datasets including the Bathybase dataset that provides field-based observations for waterbodies in the central US, bathymetry datasets from state agencies in the US waterbodies^32^, and other global resources such as the G-REALM dataset. Figure 1 shows the distribution of global waterbodies *Dmax* and location of observation waterbodies subset.

### Mapping bathymetry and development of h-A-V relationships for waterbodies in the HydroLAKES dataset

Advances in Geographic Information Systems have facilitated mapping of Earth’s surface with the capability of estimating underwater topography^4,8,33^. We used a distance method in this study to develop the bathymetric maps of the GLOBathy dataset. The distance method is a GIS-based technique and has been applied to waterbodies in the northeastern US with a wide range of geophysical conditions. Comparison of the results with field bathymetry data has shown an overall satisfactory performance and improved estimates of bathymetry and volume of the test waterbodies^8^.

**Fig. 2:**
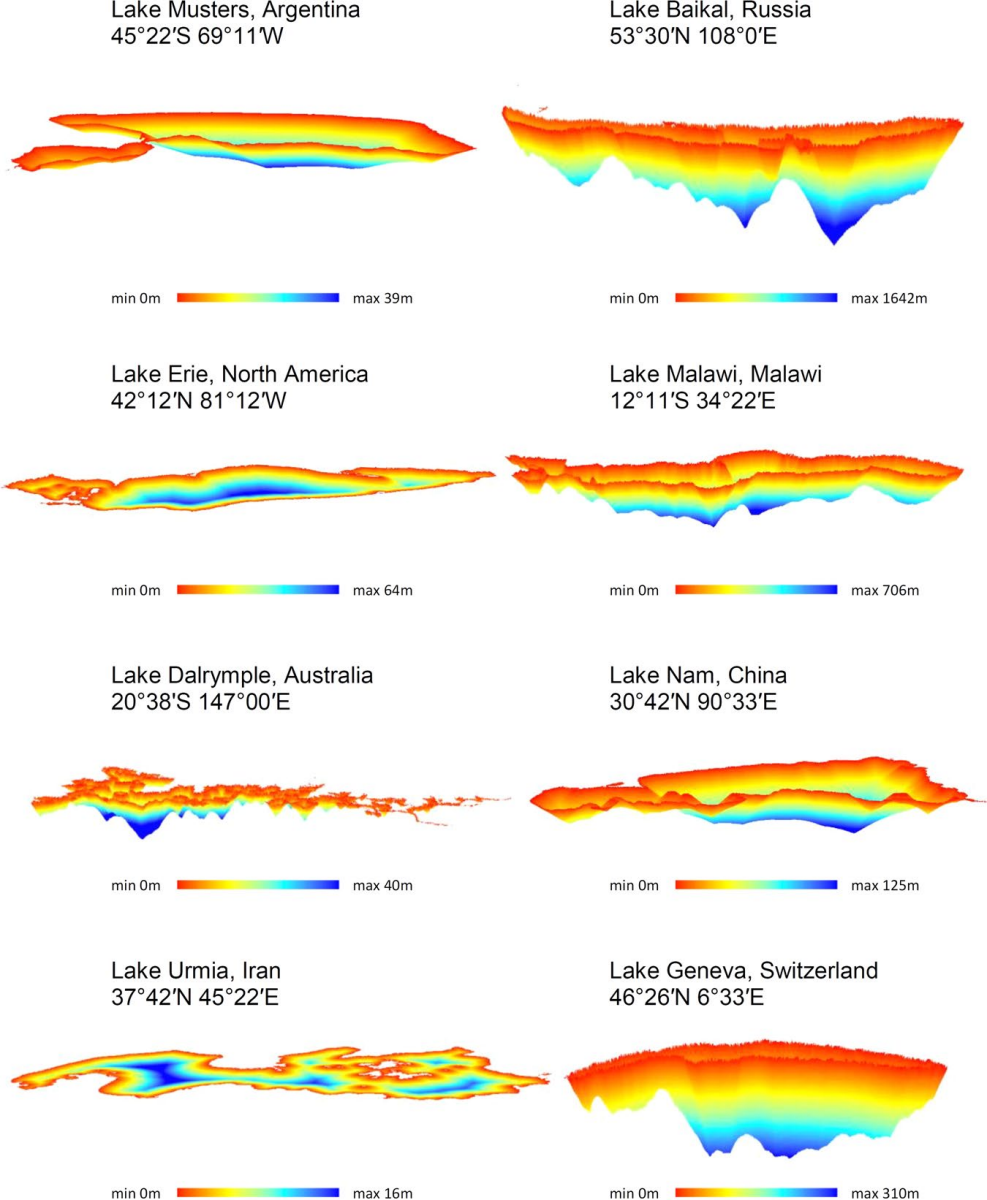
Bathymetric maps for selected waterbodies in the GLOBathy dataset.

The distance method^8^ consists of three steps: 1) convert the waterbody polygon to raster data, 2) calculate the closest Euclidean distance of each waterbody cell in the raster data to the waterbody shoreline as well as the maximum distance to the shore, and 3) use Eq. 1 to convert distance into depth (*D*) for each cell in the waterbody:

1 $$D=\frac{l\times {D}_{max}}{L}$$

where *l* is Euclidean distance of the corresponding waterbody cell to the shoreline, *Dmax* is the maximum depth of the waterbody (estimated in the previous section), and *L* is the maximum distance of the waterbody cells from the shoreline. Figure 2 shows estimated bathymetric maps for some of the selected waterbodies worldwide.

The *h-A-V* relationships were derived using bathymetric information of each waterbody in the GLOBathy dataset and take the form of polynomial functions as follows:

2 $$A=a{h}^{b}$$

3 $$V=c{h}^{d}$$

where *a, b, c*, and *d* are the unknown empirical coefficients for each waterbody, and *A* and *V* are the surface area and volume of the waterbody at water level *h* (with respect to the bottom of the waterbody). To estimate the unknown coefficients, the bathymetry was used to calculate *A* and *V* at ten depth layers evenly distributed in the vertical direction of the waterbody profile. Given values for *h, A, V*, the polynomial function was fitted to these *(h, A)* and *(h, V)* data points to find the best estimates of the empirical coefficients for both *A* = *f(h)* and *V* = *f(h)* relationships. Specific details of the bathymetric and *h-A-V* data are provided in the Data Records section.

## Data Records

The data products of the GLOBathy dataset can be obtained from the *figshare* repository^34^. These products include: 1) bathymetric maps, 2) *Dmax* estimates, and 3) *h-A-V* relationship estimates for each waterbody in the GLOBathy (and also HydroLAKES) dataset. The details of these products are provided in Table 2.

**Table 2:** Details of the GLOBathy products and data files.

Filename/Directory	Number of Data Files	Descriptions
***Bathymetry_Rasters.zip***	1,427,688	raster files of bathymetric maps in Tagged Image File Format (TIFF) for each individual waterbody in resolution of 1 arc-seconds and in WGS84 projection system
***GLOBathy_basic_parameters.zip***	17	“*GLOBathy_basic_parameters(ALL_LAKES).csv*” provides estimation of *Dmax* based on four different geometric shapes (box, cone, triangular prism, and ellipsoid) and the two empirical methods [*Dmax = f(P, A)* and *Dmax = f(P, A, V, Elev, WA)*]
		15 spreadsheets with the name pattern “GLOBathy_basic_parameters(*LAKES).csv” that each includes the same information as the “*GLOBathy_basic_parameters(ALL_LAKES).csv*” file above but in smaller csv files (100,000 waterbody increments in each file) for easier navigation of the dataset
		“*GLOBathy_basic_parameters_README.txt*” that provides details for attributes of the spreadsheets
***GLOBathy_hAV_relationships.nc***	1	estimation of *h-A-V* relationships derived from polynomial functions of *A = f(h)* and *V = f(h)* using the bathymetric maps of each waterbody in the Network Common Data Form (NetCDF) format

Waterbody parameters are abbreviated as: Dmax = maximum depth, P = shoreline length, A = surface area, V = total volume, Elev = elevation of waterbody surface, WA = area of waterbody watershed, h = water level in the waterbody (with respect to the bottom), h-A-V = head-Area-Volume relationships.

## Technical Validation

To evaluate the performance of the empirical models of *Dmax*, a set of waterbodies with observations of *P*, *A*, *V*, *Elev*, *WA*, and *Dmax* was compiled. This independent dataset of *Dmax* observations for 1,503 waterbodies are from three main sources: Bathybase; Texas waterbodies in the US by the Texas Water Development Board (TWDB; https://www.twdb.texas.gov/surfacewater/surveys/completed/list/index.asp); and the G-REALM dataset. Additionally, we used a variety of online reservoir databases to manually identify the parameters for waterbodies larger than 500 km^2^ in surface area. This observation dataset of *Dmax* was then paired with corresponding estimates of *P*, *A*, *V*, and other geophysical characteristics (i.e., waterbody surface elevation, average depth, and watershed area) of the HydroLAKES dataset. The set of validation waterbodies was constructed to represent the global distribution of waterbodies, spatially (see Fig. 1) and for a wide range of geophysical properties as shown by the summary statistics in Table 3.

**Table 3:** Summary statistics of the 1,503 observational waterbodies dataset. MCM denotes million cubic meters.

Waterbody Parameter	Average	Minimum	Maximum	Median
Shoreline length (km)	238	1.38	15828	8.91
Surface area (km^2^)	894	0.10	377002	2.10
Volume (MCM)	113455	0.24	75600000	8.91
Waterbody surface elevation (m)	406	−415.00	4724	366.00
Watershed area (km^2^)	26457	0.20	2764126	53.61
Maximum depth (m)	34.29	0.50	1642	13.10

**Fig. 3:**
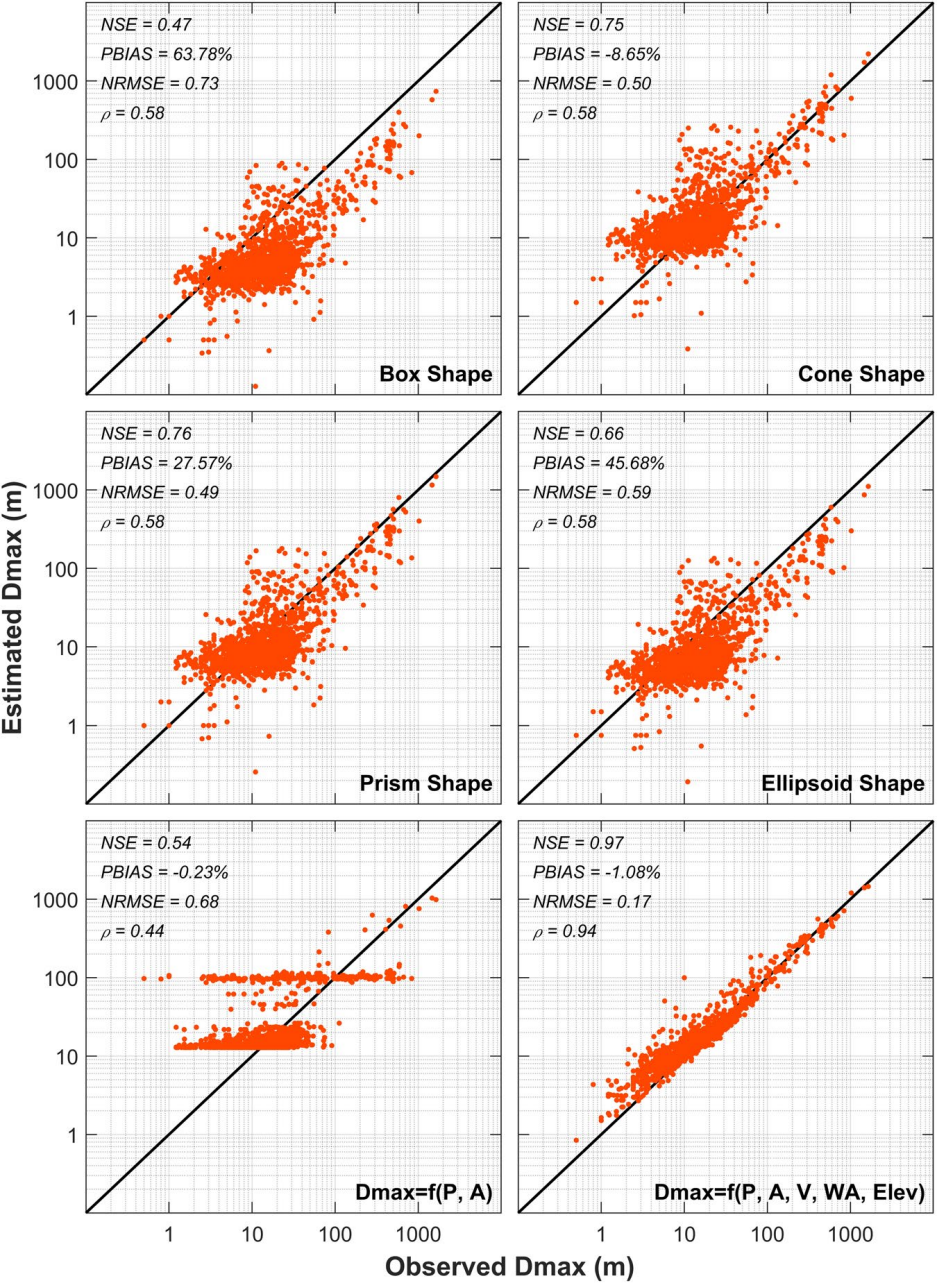
Comparison of observed vs estimated maximum depth (Dmax) based on four selected geometric shapes and two empirical relationships as a function of shoreline length (P), surface area (A), volume (V), watershed area (WA), and waterbody surface elevation (Elev).

Figure 3 illustrates the validation of observed versus estimated *Dmax* for the observational waterbodies based on the different approaches explained above (i.e., four geometric shapes and two functional forms). We selected Nash-Sutcliffe efficiency (NSE), percentage bias (PBIAS), root mean squared error normalized with standard deviation (NRMSE), and Spearman’s Rho correlation coefficient (ρ) model skill criteria to evaluate the accuracy and bias in predicting *Dmax*. As shown by the model skill coefficients, the triangular prism (NSE = 0.76, PBIAS = 27.57%, NRMSE = 0.49, and ρ = 0.58) and cone (NSE = 0.75, PBIAS = −8.65%, NRMSE = 0.50, and ρ = 0.58) shapes performed relatively well in prediction of the observational waterbodies *Dmax*.

Based on model skill evaluations, functional form *Dmax* = *f(P, A, V, WA, Elev)* guarantees predicted *Dmax* is representative of geophysical characteristics of the waterbodies. Functional form *Dmax* = *f(P, A)* estimates *Dmax* with less accuracy, however, it provides a simpler prediction framework because it estimates *Dmax* as a function of *P* and *A*, which are surface variables and available (or can be easily estimated using GIS and RS) with high accuracy for almost every waterbody on Earth’s surface.

Model skill criteria indicate that the first functional relationship suggested above, i.e., *Dmax* = *f(P, A, V, WA, Elev)*, provides a more realistic estimate of *Dmax* for observational waterbodies (NSE = 0.97, PBIAS = −1.08%, NRMSE = 0.17, and ρ = 0.94) in comparison to triangular prism and cone shape methods. This model also performs better in estimation of *Dmax* for shallow lakes compared to the other geometric and functional relationships as shown in Fig. 3. To verify the robustness of this empirical function, we carried out a random cross validation analysis in which we divided the observational waterbodies dataset into train and test subsets in 100 iterations. Then we tested the out-of-sample predictive capability of the model developed based on the train set in each iteration. Average model skill criteria based on the 100 iterations for both train (NSE = 0.91, PBIAS = 1.96%, NRMSE = 0.21, and ρ = 0.91) and test (NSE = 0.86, PBIAS = −3.18%, NRMSE = 0.29, and ρ = 0.89) subsets indicated good performance of the model. Comparison of the observed and estimated *Dmax* values based on the functional relationship developed using *P* and *A* only (NSE = 0.54, PBIAS = −0.23%, and NRMSE = 0.68, and ρ = 0.44) and average model skill in cross validation analysis for train (NSE = 0.44, PBIAS = 1.61%, NRMSE = 0.74, and ρ = 0.49) and test (NSE = 0.38, PBIAS = −4.49%, NRMSE = 0.83, and ρ = 0.40) subsets in 100 iterations do not suggest improvements in accuracy of the predicted results. That implies *P* and *A* alone, although accurately available, are not reliable to be used for *Dmax* estimation in global scales.

**Fig. 4:**
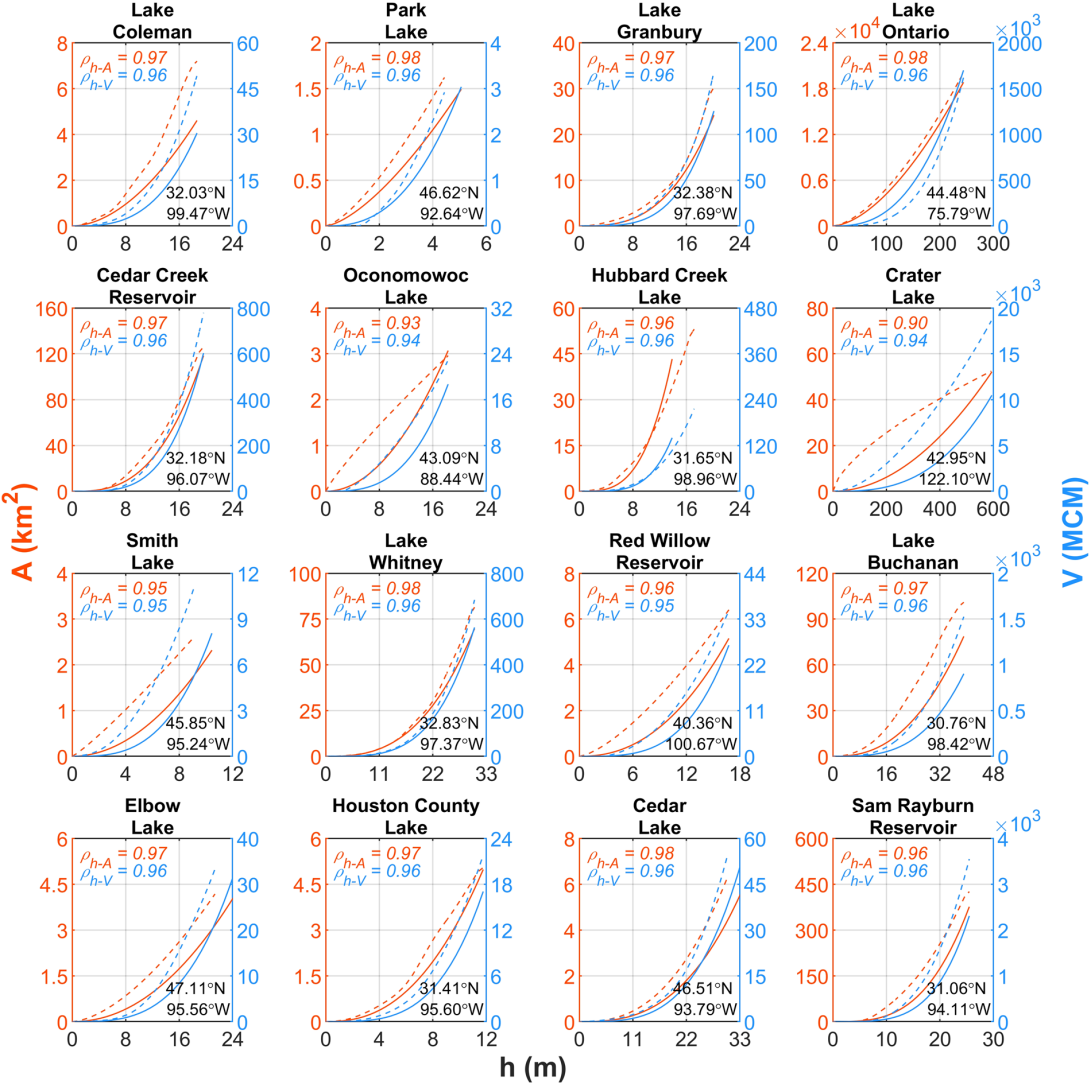
Validation of head-Area-Volume (h-A-V) estimation for selected observation waterbodies. Solid and dotted lines denote h-A-V relationships based on GLOBathy bathymetry maps and observations, respectively. Also, red and blue colors indicate h-A and h-V relationships, respectively. MCM denotes million cubic meters. Latitude and longitude values show pour point location of each waterbody.

As shown in Table 1, Texas waterbodies dataset has provided *h-A-V* estimations for several waterbodies in Texas, US through field surveys. Figure 4 shows comparison of observed and estimated *h-A-V* relationships for selected waterbodies in the Texas waterbodies dataset, as well as those inferred from ground-based bathymetry data in other locations. To represent different geophysical conditions, waterbodies of various geometric characteristics were selected for comparison. Model skill criteria shows that estimated *h-A* and *h-V* polynomial functions compare well with observed information.

Finally, to validate the quality of estimated bathymetric maps of the GLOBathy in terms of representing 2D variability in lake depth, we compared bathymetry of selected lakes in the dataset with ground-based bathymetry observations in different geographic locations. This comparison is provided in Figure S1 of the Supplementary Information. Observational bathymetry maps are limited, yet we tried to select waterbodies within a wide range of physical characteristics (e.g., shape at surface area, maximum depth, lake volume, natural/unnatural) and in different geographic locations. Figure S1 shows that GLOBathy estimates bathymetry with reasonable accuracy and resembles the patterns of depth variability for these selected waterbodies fairly well, given the complexity of estimating underwater topography.

## Usage Notes

GLOBathy can be used in a suite of applications including limnology, hydrodynamic modeling, aquatic systems chemistry and biology, hydrology, and water resources management. In particular, GLOBathy is suitable for large-scale geophysical studies (e.g., continental and global scales) where numerous waterbodies are present and observational-based data is not available for each waterbody in the system.

Existing ground-based datasets often provide bathymetry data for large or well-known waterbodies, with data not uniformly distributed or available worldwide. However, GLOBathy provides detailed depth and bathymetric information for more than 1.4 million waterbodies globally, including small lakes that are often neglected in bathymetry datasets. Those small waterbodies are important for ecosystem functioning, water supply and storage, the hydrological cycle, and in evaluating global or regional carbon-cycling processes^16,35,36^. Additionally, GLOBathy provides computational tools (*“Generate_Bathymetry_Rasters.py”* Python program; see Code Availability section for more details) to create bathymetric maps for any waterbody that is excluded from the dataset.

One major advantage of GLOBathy is that it can be used as a complementary source of information to study waterbodies or geophysical systems worldwide. For instance, it can be merged with HydroLAKES, ReGeom^17^, and GLCP^16^ to study surface water availability and the global distribution of surface water resources. It can also be used jointly with several datasets such as G-REALM, DAHITI, and Cooley *et al*.’s analysis of global waterbodies water levels^12^ to study surface water variability. GLOBathy can be utilized in water resources management studies in concert with datasets that provide information on physical attributes of reservoirs and dams such as Global Reservoir Surface Area Dataset (GRSAD)^37,38^, GranD (https://globaldamwatch.org/grand/), Reservoir Assessment Tool (RAT)^39^, GlObal geOreferenced Database of Dams (GOODD)^40^, and Georeferenced global Dam And Reservoir (GeoDAR)^41^. GLOBathy also complements datasets with hydrographic information such as National Hydrography Dataset Plus (NHDPlus; https://www.epa.gov/waterdata/nhdplus-national-hydrography-dataset-plus), HydroBASINS^42^, and HydroRIVERS^42^ (https://hydrosheds.org/) which can lead to improvements of hydrological modeling and understanding the global water cycle. For example, GLOBathy can be used in the National Oceanic and Atmospheric Administration’s (NOAA) National Water Model (NWM; https://water.noaa.gov/about/nwm) to improve assignment of reservoirs’ physical attributes and hydrological forecasting over the entire continental United States (CONUS) domain.

In addition, many ecological and biogeochemical studies of inland waterbodies depend on bathymetry and detailed depth information of aquatic systems. GLOBathy can contribute to those studies by improving the understanding of depth variations in waterbodies worldwide. In that regard, GLOBathy complements the Global Lake Ecological Observatory Network (GLEON)^36^, Lake-Catchment (LakeCat)^43^, and LAGOS (https://lagoslakes.org/) datasets^44,45^ that aim to represent water quality of inland waterbodies. Understanding physical processes in waterbodies also depends on detailed bathymetric information. Therefore, using realistic bathymetric information could lead to improvements in modeling currents, surface fluxes and evaporation, water temperature, waves, erosion and resuspension, nutrient and particle transport in waterbodies, especially for those cases with lack of ground-based bathymetric data where often simplified depth conditions are used. Weather and climate prediction models (e.g., Unified Forecast System; https://ufscommunity.org/) also depend on accurate information on waterbody surface temperature, therefore, GLOBathy could be a useful resource in that regard by improving water temperature modeling of waterbodies.

It is obvious that datasets compiled from field surveys provide more reliable bathymetry information than other sources of data. However, previous efforts to create observational bathymetry datasets are limited and only available in local scales (e.g., Bathybase, Texas and Minnesota Waterbodies, and RESSED) due to difficulties and costs of obtaining field-based underwater topography. That might leave gaps in definition of the geophysical systems in large-scale studies (where a great number of waterbodies might be present), therefore, observational data might be combined with model- and/or RS-based bathymetry such as GLOBathy to fill the gaps and refine system representation.

## Supplementary information

Supplementary Information

## Data Availability

We provide two Python scripts to accompany the GLOBathy dataset: *“Generate_Bathymetry_Rasters.py”* prepares bathymetric maps of the GLOBathy dataset. It requires two inputs: 1) a csv file containing maximum depth of the waterbodies (e.g., “*GLOBathy_basic_parameters(ALL_LAKES).csv*” can be used as a template), and 2) polygon shapefiles of the corresponding waterbodies (e.g., *“HydroLAKES_polys_v10.shp”* obtained from the HydroLAKES dataset can be used as a template). This script can be used to re-generate GLOBathy data with new *Dmax* estimations/observations or for any other case study, as long as the waterbody *Dmax* value and shapefile are available. In addition, we provide the *“WGS_84_cell_dimesion_calculator.py”* script which can be used to calculate cell dimensions of the GLOBathy raster files in South-North and East-West directions. It will provide the cell dimensions for any given location, so that accurate distances and volumes may be calculated. This is necessary because the geocentric coordinate system of the input raster data (WGS84) does not preserve distances. This script requires either the average latitude of the domain to be updated in the script header manually or a path to at least one bathymetry raster file to obtain the dimensions. In the first case the outputs are average cell dimensions of the study area. In the latter case for each bathymetry raster input, a csv file is generated that includes cell dimensions for every cell in the raster file. This script can also be used for other cases with a similar geocentric coordinate system. Script options (at the beginning of the script) need to be updated based on the input raster file.
