# Supplementary material for: GLOBathy, the global lakes bathymetry dataset
Source: Sci Data. 2022 Feb 3;9:36. doi: 10.1038/s41597-022-01132-9 (PMC8814159; doi:10.1038/s41597-022-01132-9)
Supplement: Supplementary file 1 — Supplementary Information [file 41597_2022_1132_MOESM1_ESM.docx]

### **Supplementary Information**

**GLOBathy, the global lakes bathymetry dataset**

Bahram Khazaei^1^, Laura K. Read^1^, Matthew Casali^1^, Kevin M. Sampson^1^, & David N. Yates^1^

1. Research Applications Laboratory, National Center for Atmospheric Research, Boulder, CO, 80301, USA

Corresponding author: Bahram Khazaei ([bkhazaei@ucar.edu](mailto:bkhazaei@ucar.edu))


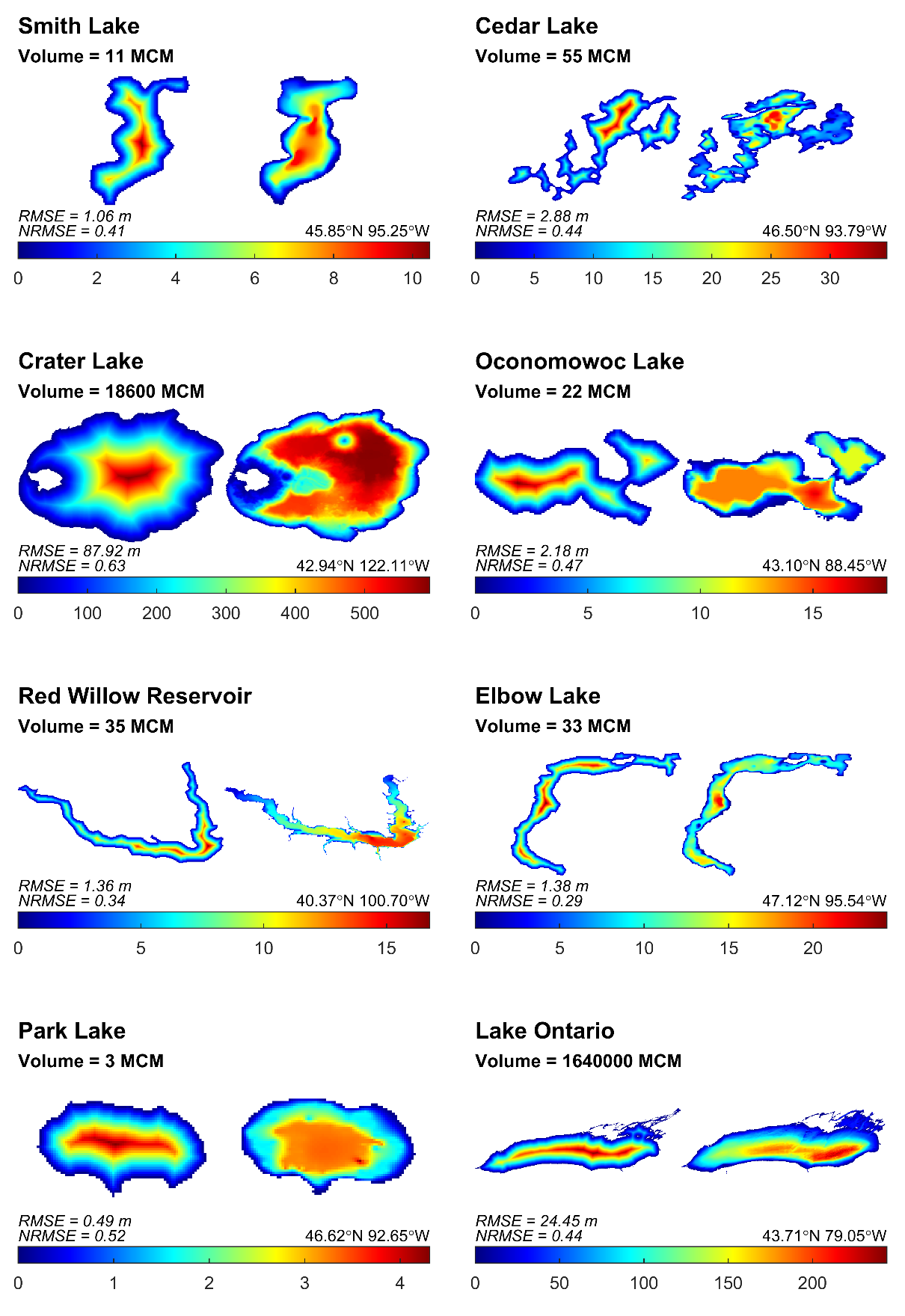


**Figure S1.** Comparison of the observed vs predicted bathymetric maps for selected waterbodies in the GLOBathy dataset. In each subplot, predicted bathymetric map from GLOBathy dataset is shown on the left side and ground-based observational bathymetric map is shown on the right side. The scale bar shows depth in m. Latitude and longitude values show waterbody pour point location.
